# Strategy to achieve mitral isthmus flutter ablation by radiofrequency: the SHERIFF plan

**DOI:** 10.1007/s10840-024-01804-z

**Published:** 2024-04-11

**Authors:** Alexis Mechulan, Pierre Dieuzaide, Angélique Peret, Thibaud Vaugrenard, Sophiane Houamria, Frederic Pons, Lyassine Nait-Saidi, Ichem Miliani, Thomas Lemann, Ahmed Bouharaoua, Sébastien Prévot

**Affiliations:** 1 Ramsay Santé, Hôpital Privé Clairval, Service Cardiologie-Rythmologie, Marseille, France; 2 Service de Cardiologie, Hôpital d’Instruction Des Armées Sainte-Anne, Boulevard Sainte-Anne, Toulon, France

**Keywords:** Perimitral atrial tachycardia, Mitral isthmus, Block line, Prospective study

## Abstract

**Background:**

Achieving mitral isthmus (MI) block can be challenging. This prospective study evaluated the feasibility and efficacy of a systematic strategy comprising three consecutive steps to achieve MI block.

**Methods:**

Twenty consecutive patients (mean (± SD) age 71.4 ± 6.98 years) undergoing ablation of perimitral atrial tachycardia (PMAT) between December 2019 and November 2021 were included. MI was ablated using a systematic strategy comprising up to three consecutive steps: (1) endocardial ablation from the superolateral mitral annulus to the left pulmonary veins; (2) additional epicardial ablation in the coronary sinus (CS) on the opposite side of the endocardial line; and (3) ablation of early activation sites between endocardial and epicardial breakthroughs.

**Results:**

MI block was successfully achieved in 19/20 patients (95%). MI block after endocardial radiofrequency ablation alone (step 1) was observed in 7/20 patients (35%). Epicardial ablation within the CS on the other side of the endocardial line (step 2) resulted in bidirectional MI block in three more patients. Endocardial ablation of epicardial conduction was successful for nine additional patients (95% success). At the 12-month follow-up, five patients (25%) displayed recurrence of arrhythmia after a single procedure. One patient had electrical cardioversion for persistent atrial fibrillation. Four patients had a redo procedure for left atrial flutter and only two patients (10%) had conduction across the MI and showed recurrence of PMAT. No complications occurred.

**Conclusions:**

The three-step ablation strategy resulted in a high rate of acute and durable MI block. PMAT recurrence after a single procedure was 10% at 1-year follow-up.

**Graphical Abstract:**

The three-step ablation strategy had a success rate of bidirectional MI conduction block of 95%. Recurrence of PMAT after a single procedure was 10% at 1-year follow-up.

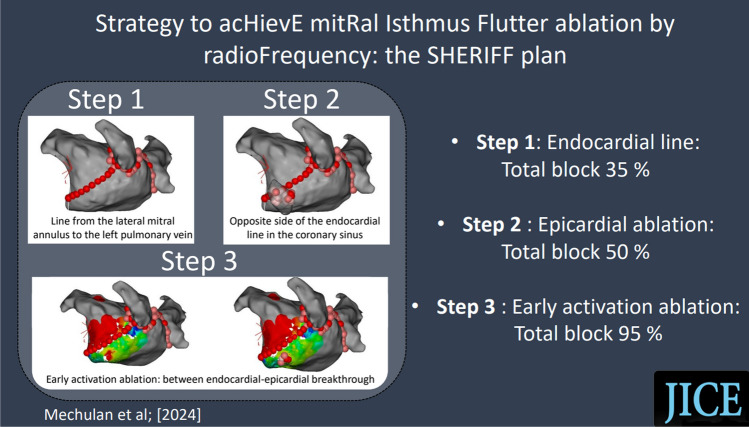

## Introduction

Successful ablation of perimitral atrial tachycardia (PMAT) using radiofrequency catheter ablation remains challenging. The standard ablation technique consists of the creation of a mitral isthmus (MI) line from the lateral mitral annulus to the ostium of the left pulmonary veins. This line is the most frequent ablation target in 42% of all cases of reentrant atrial tachycardia [1].

Achieving MI bidirectional conduction block can be technically difficult and reported success rates range from 32 to 98.2% [2–6]. Results have been highly heterogeneous between studies particularly regarding ablation technique and location of line to prevent PMAT [7, 8]. The ALINE study reported that after a single linear lesion, complete block was achieved only in 23% of patients after a first pass [9]. Low success rates can probably be explained by the anatomical characteristics of the MI region. Many patients need additional ablation within the coronary sinus (CS). One main obstacle for MI ablation is the presence of epicardial electrical connections between the left atrium and the vein of Marshall (VOM) that can bypass the MI line. For these patients, ablations are performed at the epicardial-endocardial insertion sites. Epicardial ablation inside the CS was reported to be required in up to 70% of patients [3, 4, 9–11].

To date, there is no consensus around a standardized technique. The objective of the present study was to evaluate the efficacy of a systematic strategy using radiofrequency to achieve MI bidirectional conduction block in patients with PMAT. This strategy comprised up to three steps: (1) creation of an endocardial line extending from the lateral mitral annulus to the left pulmonary vein; (2) epicardial ablation in the CS on the opposite side of the endocardial line when conduction persisted after step 1; and (3) if conduction block was still not achieved, ablation of early activation between endocardial and epicardial breakthrough locations was performed.

## Methods

### Study population

The study population included a series of 20 consecutive patients (45% female, mean (SD) age 71.4 ± 6.98 years, range 57.6–81.7 years) who underwent PMAT radiofrequency ablation at our institution (Hôpital Privé Clairval, Ramsay Santé, France). Patients who had undergone previous MI ablation were excluded. All patients gave their written informed consent before taking part in the study. The study was approved by the local institutional review board (COS-RGDS-2023–02-002-MECHULAN-A) and complied with the Declaration of Helsinki.

### Catheter ablation

All procedures were performed under general anesthesia. In the majority of patients, the procedure was performed under uninterrupted anticoagulation. Using trans-septal access guided by transesophageal echocardiography, catheters were inserted into the left atrium via irrigated sheaths (SL0 sheath and Agilis; St Jude Medical Inc., St Paul, MN, USA). 3D-electroanatomical mapping of the left atrium was performed using a navigation system (CARTO3™; Biosense Webster Inc., Diamond Bar, CA, USA) and a duo decapolar electrode catheter (Pentaray®; Biosense Webster Inc., Diamond Bar, CA, USA). A contact force catheter with an advanced irrigated porous tip (ThermoCool Smarttouch SF™; Biosense Webster Inc., Diamond Bar CA, USA) was used for ablation. Irrigating rate during ablation was 15 ml/min. 3D-electroanatomical mapping was performed after identification of P-waves on the surface electrocardiogram. The end of the window of interest was set at the end of the P-wave [12]. Atrial signal on the CS electrograms was used as the reference signal. Diagnosis of PMAT was established by activation mapping and entrainment. The ablation strategy involved MI ablation and pulmonary vein isolation (PVI) for patients that underwent a first procedure, or when reconnection was observed for patients that underwent a second or a third procedure.

### Three-step ablation

**Fig. 1:**
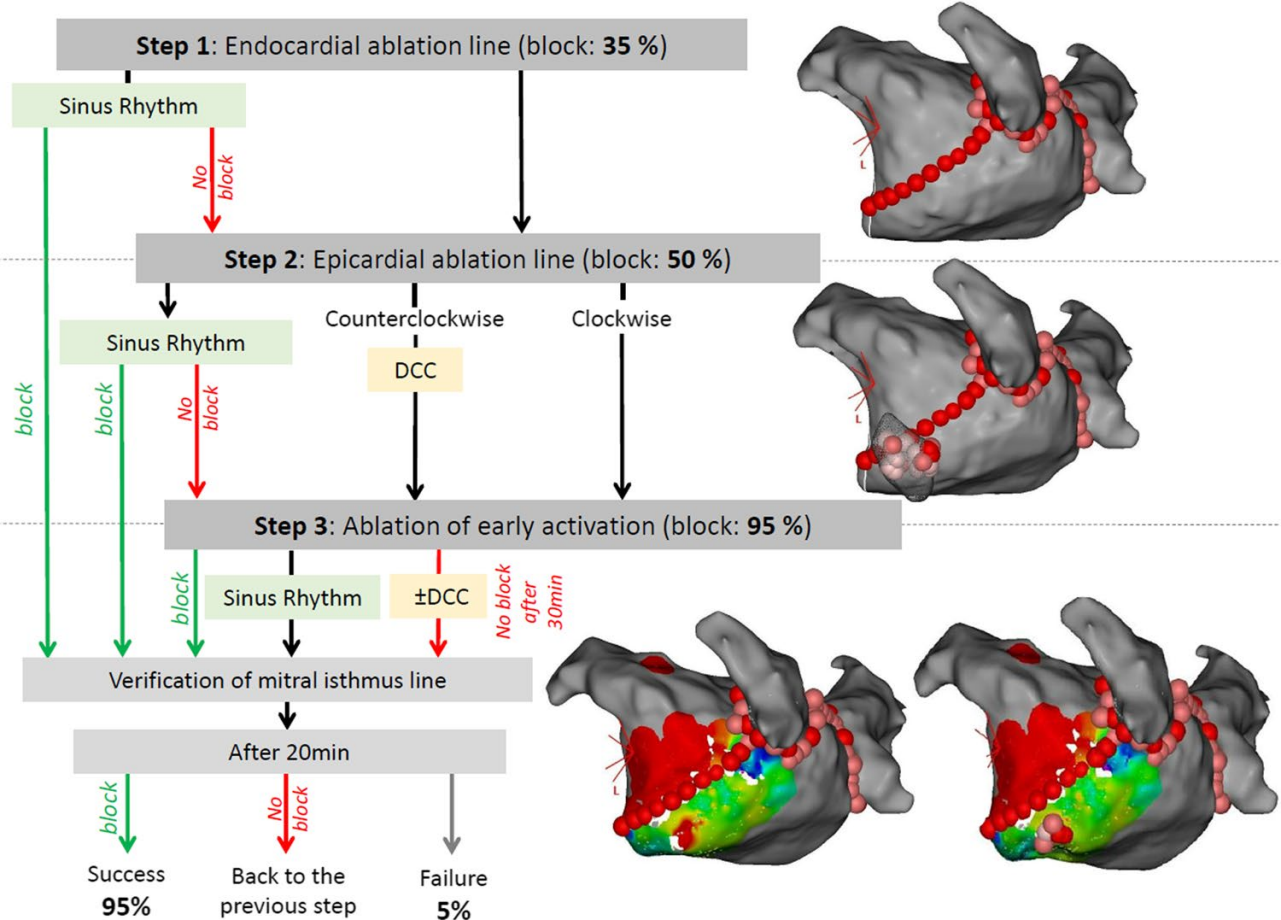
Results of the three-step strategy illustrated by left atrial 3-dimensional electroanatomical maps. DCC direct current cardioversion

Ablations were performed using a systematic three-step strategy (Fig. 1). Step 1 consisted of creating a MI line point by point using radiofrequency ablation at a power of 40 W. The ablation index (AI) was ≥ 550; ablation time was between 30 and 40 s. Algorithm Carto3 Visitag Surpoint was used to guide the ablation. The contact force used was ≥ 10 g according to the operator’s judgment. When no block or signal was recorded on the ablation line, step 2 was performed. Step 2 consisted of epicardial ablation in the CS on the opposite side of the MI line obtained after step 1, with an AI ≥ 350 and power of 20 W. The targeted areas were successfully reached in all patients. The Agilis catheter was used to reach the distal part of the CS. Direct current cardioversion was performed when counterclockwise PMAT was observed to map epicardial breakthrough under the MI. When no block was still observed, step 3 was performed. For this step, the earliest activation site (endocardial or epicardial) considered conduction breakthrough area was identified and ablated. This process was repeated until MI was block was achieved. The endpoint was bidirectional conduction block of MI. Bidirectional conduction block was reassessed 20 min after the last ablation lesion. The presence of block was confirmed if pacing from the left atrial appendage produced a proximal to distal activation on the catheter positioned in the CS.

### Follow-up

At 1-year follow-up, data were collected from patient’s cardiologist who performed an ECG and Holter or directly from the patients. Recurrence was defined as a documented episode of atrial fibrillation (AF) or atrial tachycardia (AT) lasting > 30 s after a 3-month blanking period.

### Second ablation procedure

All second ablation procedures followed the same general principles as the first. Conduction block of the multiple lines of the lesion set was assessed as described above and gaps were closed systematically.

### Statistical analysis

Descriptive variables are presented as means ± standard deviation (SD). Analyses were performed using GraphPad Prism (GraphPad software 5.01).

## Results

### Patient characteristics

The study population included 20 patients who underwent *de novo* MI line ablation between December 2019 and November 2021. The demographic characteristics of the study population are shown in Table 1. Mean age was 71.4 ± 7 years. Nine of the patients (45%) were female. Mean left atrium size was 154.1 ± 51.7 ml and left ventricular ejection fraction was 53.5 ± 8.4%. Ablation was indicated for PMAT in 12 patients (60%), for persistent atrial fibrillation (PersAF) in 7 patients (35%), and for roof-dependent atrial flutter in 1 patient (5%). Among the 20 patients, seven ablations were performed without any previous ablation procedure, 11 patients underwent their second procedure, and two underwent their third. For patients who underwent a second ablation, ten patients had a history of PersAF, one had ParoxAF and two had typical atrial flutter. For patients with prior PersAF, PVI, roof line, and ablation of complex fractionated atrial electrograms were performed. For patients with prior ParoxAF, only PVI was performed. For two patients who underwent a third ablation, the index procedure was ParoxAF ablation and typical flutter ablation. PVI and roof line were performed for patients included for a first ablation. The clinical characteristics of the patients are summarized in Table 2.

**Table 1:** Demographic and clinical characteristics of the study population at inclusion

Characteristic	Total (*N* = 20)
Age (years)	71.45 ± 6.98
Sex (female)	9 (45%)
BMI	28.74 ± 4.76
Amiodarone	12 (60%)
History of cardioversion	8 (40%)
Cardiac parameters	
LA volume (ml)	154.13 ± 51.7
LVEF (%)	53.5 ± 8.44
Comorbidities	
Diabetes mellitus	4 (20%)
Hypertension	14 (70%)
OSA	6 (30%)
Coronary artery disease	10 (50%)
CHA_2_DS_2_-VASc score	3.25 ± 2.15

Data are shown as mean ± standard deviation or *n* (%)

*BMI* body mass index, *LA* left atria, *LVEF* left ventricular ejection fraction, *OSA* obstructive sleep apnea

**Table 2:** Ablation context and 1-year follow-up

Patient no	Ablation context	Block	1-year follow-up: recurrence	Recurrence type	1-year follow-up: reconnection of MI line	Previous procedure no. 1	Procedure used in procedure no. 1	Previous procedure no. 2	Procedure used in procedure no. 2
1	PMAT	Yes	No	-	-	PersAF	CFAE + roof	ParoxAF	PVI
2	PMAT	Yes	No	-	-	PersAF	PVI + CFAE + roof	-	-
3	PMAT	Yes	No	-	-	ParoxAF	PVI + roof	Typical flutter	CTI ablation
4	PersAF	Yes	Yes	PMAT	Yes	-	-	-	-
5	PMAT	Yes	No	-	-	-	-	-	-
6	PersAF	Yes	No	-	-	-	-	-	-
7	PMAT	Yes	No	-	-	-	-	-	-
8	PersAF	Yes	No	-	-	-	-	-	-
9	PersAF	Yes	No	-	-	PersAF	PVI + CFAE + roof	-	-
10	PMAT	Yes	Yes	Typical flutter	No	PersAF	PVI + CFAE + roof	-	-
11	PersAF	Yes	No	-	-	-	-	-	-
12	PMAT	Yes	No	-	-	Typical flutter	CTI ablation	-	-
13	PersAF	Yes	No	-	-	-	-	-	-
14	PMAT	Uncertain	Yes	PMAT	Yes	PersAF	PVI + CFAE + roof	-	-
15	PersAF	Yes	No	-	-	Typical flutter	CTI	-	-
16	PMAT	Yes	Yes	PersAF	UK	PersAF	PVI + CFAE + roof	-	-
17	PMAT	Yes	No	-	-	PersAF	PVI + CFAE + roof	-	-
18	Roof-dependent atrial flutter	Yes	No	-	-	PersAF	PVI + CFAE + roof	-	-
19	PMAT	Yes	No	-	-	PersAF	PVI + CFAE + roof	-	-
20	PMAT	Yes	Yes	mAT of LAA	No	PersAF	PVI + CFAE + roof	-	-

*PMAT* perimitral atrial tachycardia, *PersAF* persistent atrial fibrillation, *ParoxAF* paroxysmal AF, *PVI* pulmonary vein isolation, *CFAE* complex fractionated atrial electrograms, *CTI* cavotricuspid isthmus, *UK* unknown, *mAT* macroreentrant atrial tachycardia, *LAA* left atrial appendage

### Ablation procedure and three-step strategy

The mean duration of the three-step strategy was 115.8 ± 25.6 min, with a radiofrequency time of 19.5 ± 6.2 min, a mean fluoroscopy duration of 2.95 ± 4.8 min, and a mean dose of 0.575 ± 0.78 mGy∙cm^2^ (Table 3). The first step consisted of creating a MI endocardial line using point-by-point ablation (Fig. 1). The mean length of the MI line was 35.3 ± 11.1 mm. After completion, blocks were verified and achieved in 7/20 patients (35%). When complete block was not obtained by endocardial ablation only, the second step was initiated. Bidirectional block using epicardial ablation was achieved for three additional patients. When conduction block could not be achieved, then the third step was completed. This step was achieved in nine patients (45%). Using this three-step strategy, 19/20 (95%) patients had bidirectional MI block. For one patient, block was uncertain despite completing all steps. One operator was uncertain of MI block despite proximal to distal activation on the CS catheter because of a short delay between CS 3–4 and 1–2. Block was obtained from the endocardium for 12 patients (60%) and within the CS for seven patients (35%). Overall block was obtained for 19 patients (95%). No complications occurred. No left atrial appendage isolation was observed.

**Table 3:** Procedural characteristics and outcomes

Characteristics	Total (*N* = 20)
Procedural characteristics	
Procedure duration (min)	115.8 ± 25.6
RF duration (min)	19.5 ± 6.2
Fluoroscopy duration (min)	2.95 ± 4.8
Fluoroscopy dose (mGy∙cm^2^)	0.575 ± 0.78
Length of MI line (mm)	35.3 ± 11.1
Block location	
Step 1	7 (35%)
Step 2	3 (15%)
Step 3	9 (45%)
Uncertain	1 (5%)
Endocardial block	12 (60%)
Epicardial block	7 (35%)
Post-procedural characteristics and outcome	
Follow-up period (months)	12.3 ± 1.65
Recurrence	5 (25%)
AT recurrence	4 (80%)
AF recurrence	1 (20%)
Recovery of conduction	2 (10%)

Data are shown as mean ± standard deviation or *n* (%)

*MI* mitral isthmus, *AF* atrial fibrillation, *AT* atrial tachycardia

### One-year follow-up

At a mean follow-up of 12.3 ± 1.7 months, 15 patients (75%) maintained their sinus rhythm and remained free from AT/AF. During follow-up, five patients (25%) suffered recurrences. Four patients had a redo procedure and one had direct current cardioversion for PersAF. Among these four patients, two had persistent MI block (one had typical atrial flutter and the other displayed AT around the left atrial appendage) and two had PMAT (10%). MI block was obtained for the two patients with PMAT recurrence. Of note, for one of the two patients, the block was uncertain at the end of the index procedure.

## Discussion

In this study, our three-step ablation strategy for bidirectional MI conduction block had a success rate of 95%. Moreover, PMAT recurrence after a single procedure was only 10% at 1-year follow-up. These findings support the effectiveness and durability of these linear lesions.

Bidirectional block in MI is mandatory but is challenging to obtain. Incomplete block is reported to increase the risk of PMAT, emphasizing the importance of achieving complete electrical block of the MI [13]. However, the results in the literature concerning the percentage of MI conduction block are extremely variable ranging from 32 to 98.2% [2–6]. In each of these studies, the techniques used were heterogeneous. The ALINE study described a protocol using ablation index to obtain homogenous lesions. The authors showed that after a first pass, block was achieved in 23% of patients and that after additional endo- and epicardial radiofrequency ablation, bidirectional MI block was observed in 80% of patients [9]. More recently, ethanol infusion of the VOM has been shown to be more effective than radiofrequency in achieving MI block. Using the Marshall-PLAN ablation strategy, Derval et al. observed that bidirectional block was achieved at the MI in 95% of patients. In the VENUS study, the authors showed that compared with catheter ablation alone, catheter ablation associated with VOM ethanol infusion increased the success rate of achieving perimitral block from 51.3 to 80.6% [14]. A novel non-thermal energy source was developed to achieve lesion formation using alternating high electrical fields applied to cardiac tissue [15]. Compared with thermal energies, the pulsed field ablation carries a tissue-specific effect, targeting cardiomyocytes. A recent study realized by Davong et al. evaluated feasibility and safety of MI ablation using PFA and demonstrated that acute MI block could be achieved in 100% of patients [16]. However, the durability of the lesions has not yet been established. Although the results obtained with VOM ethanol infusion and PFA are encouraging, the limits displayed and safety are still open to debate. In the current study, only radiofrequency ablation was used to control the extent of the lesion and thus to provide a reproducible approach.

In addition to the development of new ablation techniques, the optimal location of the ablation line was also studied. Ammar et al. described a modified anterior line connecting the anterior/anterolateral mitral annulus with the left superior pulmonary vein [8]. In the present study, MI line block was achieved using the superolateral line described by Maurer et al. [7]. In fact, these authors observed a high success rate of 98.2%. However, in our study, epicardial ablation was required in 60% of patients compared to 7% [7]. Our results are nevertheless consistent with the literature as in the study of Sakamoto et al., endocardial block was initially obtained in only 18% of patients [17]. Our three-step approach requires the creation of an additional map to identify epicardial conduction. Although it has been described in the literature, targeting epicardial conduction by endocardial or within the CS radiofrequency ablation was not reported systematically. In our study, this third step was necessary in 45% of patients to achieve MI block. The advantage of this approach is its reproducibility and precise control of the extent of the lesions. To date, radiofrequency ablation remains the gold standard technique and its safety and efficiency have already been demonstrated.

## Limitations

The main limitation of this study was the small sample size and the single-center study design. A longer duration of monitoring may be necessary to evaluate maintenance of sinus rhythm and durability of MI block.

## Conclusion

In conclusion, our three-step ablation strategy had an acute success rate of 95% to achieve bidirectional MI conduction block. Moreover, at 1-year follow-up, recurrence of PMAT after a single procedure was only 10%. These findings support the effectiveness and durability of this strategy.

## Data Availability

Data is available on request.
